# NGS Techniques Reveal a High Diversity of RNA Viral Pathogens and Papillomaviruses in Fresh Produce and Irrigation Water

**DOI:** 10.3390/foods10081820

**Published:** 2021-08-06

**Authors:** Marta Itarte, Sandra Martínez-Puchol, Eva Forés, Ayalkibet Hundesa, Natàlia Timoneda, Sílvia Bofill-Mas, Rosina Girones, Marta Rusiñol

**Affiliations:** 1 Laboratory of Viruses Contaminants of Water and Food, Department of Genetics, Microbiology and Statistics, Faculty of Biology, University of Barcelona, 08028 Barcelona, Spain; mitarte@ub.edu (M.I.); smartinezpuchol@ub.edu (S.M.-P.); efores@ub.edu (E.F.); ahundesa@ub.edu (A.H.); sbofill@ub.edu (S.B.-M.); 2 The Water Research Institute (IdRA), University of Barcelona, 08001 Barcelona, Spain; 3 Department of Marine Biology and Oceanography, Institute of Marine Science, Consejo Superior de Investigaciones Científicas (CSIC), 08003 Barcelona, Spain; timoneda@icm.csic.es; 4 Institute of Environmental Assessment & Water Research (IDAEA), Consejo Superior de Investigaciones Científicas (CSIC), 08034 Barcelona, Spain; marta.rosinol@idaea.csic.es

**Keywords:** organic food, irrigation water, viral pathogens, food safety, next-generation sequencing, target enrichment sequencing, amplicon deep sequencing, human papillomavirus, norovirus, vertebrate viruses

## Abstract

Fresh fruits and vegetables are susceptible to microbial contamination at every stage of the food production chain, and as a potential source of pathogens, irrigation water quality is a critical factor. Next-generation sequencing (NGS) techniques have been flourishing and expanding to a wide variety of fields. However, their application in food safety remains insufficiently explored, and their sensitivity requires improvement. In this study, quantitative polymerase chain reaction (qPCR) assays showed low but frequent contamination of common circulating viral pathogens, which were found in 46.9% of samples of fresh produce: 6/12 lettuce samples, 4/12 strawberries samples, and 5/8 parsley samples. Furthermore, the application of two different NGS approaches, target enrichment sequencing (TES) for detecting viruses that infect vertebrates and amplicon deep sequencing (ADS), revealed a high diversity of viral pathogens, especially Norovirus (NoV) and Human Papillomavirus (HPV), in fresh produce and irrigation water. All NoV and HPV types found in fresh fruit and vegetable samples were also detected in irrigation water sources, indicating that these viruses are common circulating pathogens in the population and that irrigation water may be the most probable source of viral pathogens in food samples.

## 1. Introduction

Consumption and production of fresh fruits and vegetables have increased over the last few years [1] due to population growth, changes in human lifestyles and growing awareness of the benefits of these foods as important sources of nutritional compounds in a healthy and balanced diet [2,3,4]. This increase in fresh food consumption, often eaten raw or minimally processed, has also been associated with an increase in foodborne infections and disease outbreaks, most of which have been linked to viral origins [2,5,6]. Pathogen contamination can occur at any stage of the food production process, from farm to fork, and irrigation water quality is a critical factor, since it is a potential source of foodborne pathogens [7,8,9,10], especially if it comes in direct contact with the edible portion [11]. The microbial quality of irrigation water is affected by a wide range of agricultural, wildlife, and human factors, including: growing season, geographical location, land use, surrounding activities, and environmental conditions [12,13,14]. Agricultural farms obtain irrigation water from sources such as reservoirs, rivers and groundwaters, and in the context of the circular economy, reclaimed water is increasingly becoming an important water source for irrigation.

In 2019, Machado-Moreira et al. collected data from several publications and established that most reported outbreaks related to ‘ready-to-eat’ food between 1980 and 2016 were due to the consumption of leafy green vegetables, including lettuce and parsley, whereas strawberries ranked first place when considering the number of cases linked to the consumption of a particular foodstuff [15]. This review also reported that leafy green vegetables and soft fruits are, in fact, the main foodstuffs implicated in the transmission of Norovirus (NoV). This virus, excreted in human feces and transmitted by the fecal–oral route, is the leading cause of reported foodborne disease outbreaks and is the main cause of viral gastroenteritis in people of all ages worldwide [5,16,17,18,19]. Other enteric viruses, such as Human Adenovirus (HAdV), Rotavirus (RoV), Hepatitis A Virus (HAV), Hepatitis E Virus (HEV), and Astrovirus (Ast), are also important agents in foodborne outbreaks [7,14,20]. Most pathogenic microorganisms of fecal origin that may be present in irrigation water cause gastroenteritis or acute hepatitis, but other pathologies—such as meningitis, myocarditis, and neurological disorders—are also possible [5].

Human papillomavirus (HPV) infects the skin and mucosal epithelia, with effects ranging from benign lesions, such as common warts, to malignant carcinomas, and its occurrence has been described in raw sewage and river waters [21,22,23,24], including high- and low-risk oncogenic HPV types [21,24]. In fact, HPV excretion in the feces of patients with diarrhea has been described, suggesting transmission through fecal shedding of a virus that was believed to be mainly epitheliotropic and, therefore, pointing to possible transmission through contaminated water [23,25].

In most foodborne viral outbreaks, the link between the contaminated food consumed and the people infected is often not easily established, which makes it difficult to intervene and implement preventive measures [26]. Current guidelines on microbiological irrigation water quality and safety from the European Union rely only on the use of *Escherichia coli* (EC) as Fecal Indicator Bacteria (FIB) [27,28]. However, it is well established that these indicators do not always correlate with important waterborne pathogens that may be present in diverse water sources, such as irrigation water [5,29,30]. This is particularly relevant for viral pathogens, which are more resistant to water treatments than bacteria, and thus, FIB might not accurately represent viral inactivation [31,32,33]. A more suitable indicator for viral fecal contamination is HAdV since it is shed in high concentrations and does not show seasonal variability [34]. HAdV is widely used as a viral fecal indicator; it is highly stable under many environmental conditions and disinfection treatments [29,35,36,37].

Quantitative polymerase chain reaction (qPCR) is the most commonly used method for the quantification of viruses in food [26], and standardized assays for NoV and HAV (ISO 1526-1:2017, https://www.iso.org/standard/65681.html, accessed on 8 April 2021) have been established [38]. However, the detection of viruses in some food matrices can be difficult due to the presence of inhibitory substances that may impact qPCR detection, leading to false-negative results [39,40,41,42]. Next-generation sequencing (NGS) is a promising tool with clear applicability to the detection of viral pathogens in the field of food safety [5,43,44,45]. The introduction of NGS techniques to this field allows the simultaneous analysis of myriad viral pathogens in a single assay, including pathogens initially not suspected to be present. This is achieved by identifying viral sequences in a sample and comparing them with established sequences in databases [39]. Therefore, NGS has great potential as a viral surveillance tool in the food production chain due to its sensitivity, broad detection range, and detailed information about the detected virus [46,47]. Despite this potential, NGS has still not been widely explored in food safety studies, and technical optimization is needed since there are limitations associated with the presence of inhibitory substances [5]. Several viral metagenomic studies applying NGS techniques in the context of food safety have been published [45,48,49,50,51,52,53], but to our knowledge, only a few have focused on fresh produce: specifically, frozen berries [54], strawberries [39], lettuce [43], parsley [5], and celery [55].

In this study, lettuce, strawberry, and parsley samples grown under organic agricultural practices as well as water irrigation sources were analyzed by qPCR for the presence and concentration of HAdV, which was used as a fecal viral indicator, and other relevant pathogens, such as NoV GI, NoV GII, and HEV. In addition, two different NGS approaches, target enrichment sequencing (TES) for the analysis of viruses that infect vertebrates and amplicon deep sequencing (ADS) for NoV and HPV, were applied to the studied samples to explore the potential application of NGS techniques for viral detection, characterization, and discovery, especially in fresh fruits and vegetables.

## 2. Materials and Methods

### 2.1. Organic Food and Water Samples

Lettuce (*n* = 12), strawberry (*n* = 12) and parsley (*n* = 8) samples were directly purchased from three different organic agriculture producers located in the province of Barcelona (Production Sites 1, 2, and 3 in Table 1), all of which are regulated by Commission Regulation (EC) No. 834/2007, No. 889/2008, and No. 1235/2008 and authorized by Consell Català de la Producció Agrària Ecològica (CCPAE). The irrigation water sources at these production sites were also sampled: 50 L of groundwater samples (*n* = 8) and 50 L of river water (flow of 0.48 m^3^/s) samples (*n* = 4). Fresh produce and irrigation water samples from organic agriculture producers were collected in July 2019.

Barcelona, with nearly 3.5 million inhabitants, has an important agricultural area on the outskirts of the city that uses water from the Llobregat river for irrigation. This river, which flows at 19 m^3^/s over a densely populated area (4948 km^2^), receives the effluents of more than 50 wastewater facilities and is affected by farming and agriculture activities. Agricultural fields at the Llobregat river delta cover an area of 3489 ha, and organic practices that use animal manure are increasing every year. Llobregat river water samples of 50 L (*n* = 2) were collected in autumn before the irrigation water intake (Table 1). Two treated wastewater samples (10 L), collected in winter, were also analyzed in this study, as this type of water is becoming an important source for irrigation, and it is also one of the main inputs of water from small river basins in the studied area (Table 1). The selected wastewater treatment plant (WWTP) is a conventional plant serving 147,000 inhabitants; it applies activated sludge as secondary treatment and a tertiary treatment based on *Phragmites australis*, a common species of reed that is autochthonous to the region and typically used in constructed wetlands and stream restoration actions [56], enabling the treatment of the secondary effluent in subsurface water flow paths receiving treated wastewater [57]. Secondary effluent (*n* = 1) and subsurface water flow-path effluent (*n* = 1) samples were collected, transported at 4 °C and processed on the same day of collection.

### 2.2. Viral Concentration and Nucleic Acid Extraction

The leafy sections of lettuce and parsley were cut into pieces with a length of approximately 2 cm and grouped into 25 g samples according to ISO 15216-1:2017 [38]. Each sample was washed for 20 min at 60 rpm in a rocker platform in Whirl-Pak^®^ plastic bags (Nasco, Fort Atkinson, WI, USA) containing 40 mL of Tris–Glycine–Beef Extract Buffer (pH 9.5, 0.25 N), and 0.25 volumes of 5× polyethylene glycol/NaCl solution was added. After precipitation for 1 h at 4 °C and centrifugation (10,000× *g*, 30 min, 4 °C), the resulting pellet was resuspended in 500 μL of PBS. Viral concentrates were treated with Turbo DNase (Invitrogen, Carlsbad, CA, USA) for 1 h at 37 °C to remove free DNA prior to nucleic acid extraction, and 500 μL of the DNase-treated viral concentrate was extracted using the NucliSENS^®^ easyMAG system (BioMérieux, Marcy l’Etoile, France). Nucleic acids were eluted in 100 μL and stored at −80 °C for further analysis in qPCR and NGS assays.

In accordance with ISO 15216-1:2017 [38], 25 g of strawberries were treated with tris–glycine–beef extract buffer and pectinase at a pH of 9.5. The solution was centrifuged (8000× *g*, 10 min, 4 °C), and the pH was adjusted to 7.0 (±0.2) by using 0.1 N HCl. Then, 0.25 volumes of 5× polyethylene glycol/NaCl solution were added. After precipitation for 1 h at 4 °C and centrifugation (10,000× *g*, 30 min, 4 °C), the resulting pellet was resuspended in 500 μL of PBS and washed with chloroform/butanol. Nucleic acids were extracted from the resulting supernatant using the NucliSENS^®^ easyMAG system (BioMérieux, Marcy l’Etoile, France) and stored at −80 °C for further analysis in qPCR and NGS assays.

Viruses were concentrated from irrigation water samples by ultrafiltration using the Long Volume Concentration kit (LVC kit) from InnovaPrep^®^ (InnovaPrep, Drexel, MO, USA) that couples to a Rexeed 25-A polysulfone hollow-fiber ultrafilter. The elution step was performed using wet foam elution cans with Tris-PBS from InnovaPrep^®^ (InnovaPrep, Drexel, MO, USA) and the eluted volume (30–50 mL) was further concentrated using Amicon Ultra-15 devices (50 kDa MWCO) from Millipore (Millipore, Burlington, MA, USA). Viral particles were recovered in a final volume of 200–500 μL of eluate and stored at −80 °C until further use. Viral concentrates were treated with Turbo DNase (Invitrogen, Carlsbad, CA, USA) for 1 h at 37 °C to remove free DNA prior to nucleic acid extraction, and 280 μL of the DNase-treated viral concentrate was extracted using the QIAamp^®^ Viral RNA Mini Kit from QIAGEN (QIAGEN, Germantown, MD, USA). Nucleic acids were eluted in 80 μL and stored at −80 °C for further analysis in qPCR and NGS assays.

### 2.3. Viral Quantification

To evaluate the level of human fecal contamination in fresh food and irrigation water samples, a specific quantitative polymerase chain reaction (qPCR) assay for HAdV was performed using TaqMan^®^ Environmental Master Mix 2.0 (Applied Biosystems, Waltham, MA, USA) with the specific primers and probe previously described [58]. Viral RNA pathogens were also quantified in all samples using the RNA UltraSense™ One-Step qRT-PCR System (Applied Biosystems, Waltham, MA, USA) with specific primers and probes for NoV GI [59,60,61], NoV GII [62,63], and HEV [64]. All assays were carried out using the Stratagene MX3000P sequence detector system (Agilent Technologies, Santa Clara, CA, USA). The qPCR standards were prepared using synthetic gBlocks^®^ Gene fragments (IDT, Coralville, IA, USA), and serial dilutions were quantified using Qubit 3.0 dsDNA HS Assay Kit (Invitrogen, Carlsbad, CA, USA). All qPCR assays were performed in quadruplicate and included non-template controls.

#### LOD Determination

The limit of detection (LoD) of the qPCR was calculated by running six 10-fold dilutions of target DNA/RNA suspensions around the detection end points (2.5, 5, 25, and 50 Genome Copies (GC)/reaction) for each analyzed virus. The concentration that produced at least 95% positive replicates was assumed to be the LoD of the qPCR assay, which was transformed to the LoD of the entire process using the sample volume or grams tested in each analysis performed.

### 2.4. Target Enrichment Sequencing (TES)

#### 2.4.1. Sequence-Independent, Single-Primer Amplification (SISPA)

A selection of samples that showed the presence of fecal contamination or specific viral pathogens were further analyzed using the TES approach, together with a negative control. Sample preparation prior to library construction consisted of the random tagging of nucleic acids and pre-amplification, allowing the study of both RNA and DNA viruses. This approach was performed following the procedure described previously [21,65,66], with the difference that SuperScript IV enzyme (Invitrogen, Carlsbad, CA, USA) was used in the retrotranscription step. Briefly, retrotranscription was performed using a random nonamer primer and followed by second-strand synthesis using Sequenase 2.0 (Applied Biosystems, Waltham, MA, USA). To obtain enough dsDNA for library construction, nucleic acids were amplified by 25 PCR cycles using AmpliTaqGold (Applied Biosystems, Waltham, MA, USA). The obtained PCR products were cleaned and concentrated with Zymo DNA Clean & Concentrate kit (Zymo Research, Irvine, CA, USA) and quantified using the Qubit 3.0 dsDNA HS Assay Kit (Invitrogen, Carlsbad, CA, USA).

#### 2.4.2. Library Construction

For each sample, libraries were constructed using the KAPA HyperPlus Library Preparation Kit (KAPA Biosystems, Roche, Basel, Switzerland). Briefly, library construction consisted of fragmentation, indexation with KAPA Dual-Indexed Adapters (KAPA Biosystems, Roche, Basel, Switzerland) and amplification of the dsDNA obtained from SISPA. Afterwards, the resulting libraries were quantified using the Qubit 3.0 dsDNA HS Assay Kit (Invitrogen, Carlsbad, CA, USA).

#### 2.4.3. Capture of Viral Sequences by VirCapSeq-VERT Capture Panel

Libraries were equimolarly pooled and captured using the VirCapSeqVERT Capture Panel (Roche, Basel, Switzerland). This panel involves the hybridization of probes designed to capture sequences from vertebrate viral pathogens, and it has enabled the detection of viral sequences in complex sample types in previous studies [21,49,50,67,68]. After the capture, quality and concentration were checked, and captured libraries were sequenced using an Illumina MiSeq 2 × 300 bp platform.

#### 2.4.4. TES Bioinformatic Processing

Paired-end FASTAQ files generated from sequencing were analyzed using Genome Detective Virus Tool Version 1.126, a web-based software used to identify, assemble, and classify all known viruses present in NGS data (https://www.genomedetective.com/app/typingtool/virus/, accessed on 4 May 2021) [69]. For more precise and accurate taxonomic classification, human viral contigs obtained with nucleotide identity above 70% were further processed and queried for sequence similarity using BLASTN against the NCBI GenBank nucleotide collection database [70,71] with Geneious R9.1.8 (https://www.geneious.com/, accessed on 4 May 2021) [72].

NCBI Taxonomy standards were followed for the species nomenclature and classification. For specific typing of human caliciviruses, the obtained NoV contigs were further analyzed using the Noronet web-based Typing Tool (version 2.0) developed by RIVM using the updated classification of NoV genogroups and genotypes [73,74,75].

### 2.5. Amplicon Deep Sequencing (ADS)

#### 2.5.1. Amplicon Generation

A selection of samples that showed the presence of fecal contamination or specific viral pathogens were further analyzed using an ADS approach. Sample nucleic acid suspensions were processed by ADS using specific nested PCR for NoV and HPV, previously described as suitable for typing purposes [21,52,76,77,78,79], with the incorporation of Illumina adapters in the nested primers. The obtained amplicons were purified from agarose gel using QIAquick Gel Extraction (QIAGEN, Germantown, MD, USA) and sequenced with an Illumina MiSeq 2 × 300 bp platform.

#### 2.5.2. ADS Bioinformatic Processing

Sequences generated from ADS were classified using BLASTN [71] (identity > 90%; coverage > 80%; length alignment: NoV > 200 bp, HPV > 100 bp) against a custom database populated with NoV prototype strain sequences from GenBank for the 9 genotypes of genogroup GI and 26 genotypes of genogroup GII determined by VP1, following the NoV classification proposed by Chhabra et al., 2019 [73] (Corrigendum 2020 [74]). The HPV database included sequences obtained from the International HPV Reference Center of Karolinska Institutet (https://www.hpvcenter.se/, accessed on 4 May 2021), consisting of all currently described HPV types, from HPV-1 to HPV-227. Other vertebrate papillomavirus genomes obtained from the Papillomavirus Episteme (PaVE) (https://pave.niaid.nih.gov/, accessed on 4 May 2021) were also included in the database.

## 3. Results

### 3.1. Virus Quantification in Irrigation Water and Organic Food

Samples of fresh fruits and vegetables from three organic farmers and irrigation water were tested for the presence of viral contaminants by specific qPCR assays. Low levels of contamination with human pathogenic viruses were detected in 46.9% of fresh produce samples and 50% of irrigation water samples (Table S1). The principal agent detected was NoV GII. Llobregat river water and treated wastewater samples showed simultaneous contamination with HAdV, NoV GI, and NoV GII, which were present in higher concentrations than in the rest of the samples.

**Figure 1:**
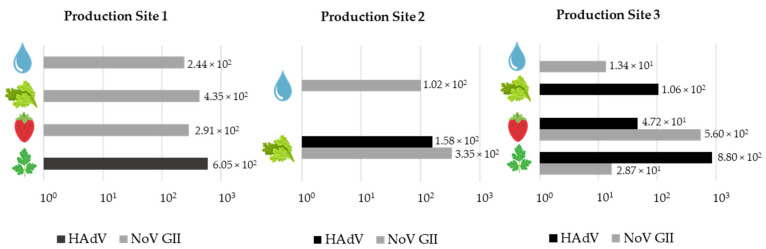
Quantification of viral pathogens in irrigation waters and harvested food samples (lettuce, strawberry, and parsley) from organic Production Sites 1, 2, and 3. Values on the X-axis show the mean concentrations of HAdV and NoV GII detected by qPCR assays, expressed in GC/25 g for food samples and in GC/L for irrigation water samples.

The presence of HAdV, an indicator of human fecal contamination, was detected in 100% of the river water samples from the Llobregat river (LLRIV.1 and LLRIV.2) and 100% of the treated wastewater samples (SE and WFPE). Lower percentages of human fecal contamination were detected in the organic food samples: 37.5% of parsley samples, 33.3% of lettuce samples and 16.6% of strawberry samples analyzed from all production sites. No human fecal contamination was detected in groundwater samples using HAdV quantification. Figure 1 summarizes the mean viral concentrations measured in irrigation waters and harvested food samples from organic production sites by specific qPCR, and Table 2 specifies viral concentrations in alternative irrigation water sources. Further details can be found in Supplementary Materials, Table S1.

NoV GI was found in 100% of Llobregat river water samples and 100% of treated wastewater samples, but NoV GI contamination was not detected in any of the irrigation water or food samples from the organic production sites. A different distribution was observed for NoV GII, which was widely present in all types of samples. This human viral pathogen was found in all irrigation water sources, including 50% of groundwater and river water samples, with the highest concentration detected in the Llobregat river sample. NoV GII was also detected in 25% of the organic food samples, with especially relevant values in all parsley samples collected from Production Site 3. HEV was not detected in any of the samples analyzed in this study.

The detection limits of the assays analyzing food samples were 75 GC/25 g for HAdV, 144 GC/25 g for NoV GI, 1036 GC/25 g for NoV GII, and 1000 GC/25 g for HEV. For the irrigation water samples, the detection limits of the assays analyzing groundwater and river water samples were 2.14 GC/L for HAdV, 4.11 GC/L for NoV GI, 29.60 GC/L for NoV GII, and 28.57 GC/L for HEV. In the analysis of treated wastewater samples, the limits of detection were 10.71 GC/L for HAdV, 20.57 GC/L for NoV GI, 148 GC/L for NoV GII, and 142.86 GC/L for HEV.

### 3.2. Virome of Irrigation Water and Organic Food Using TES

A selection of samples that showed the presence of fecal contamination or specific viral pathogens were further analyzed using the TES approach. These were: GW1.1, STR1.1, GW2.1, RIV3.1, LET3.4, PAR3.2, LLRIV.1, SE, and WFPE. This approach consisted of capturing sequences from vertebrate viral pathogens during library preparation using the VirCapSeqVERT Capture Panel (Roche, Basel, Switzerland). TES enabled the detection of human and other vertebrate viruses in all irrigation water samples and the identification of a human virus belonging to *Caliciviridae* in a parsley sample.

**Figure 2:**
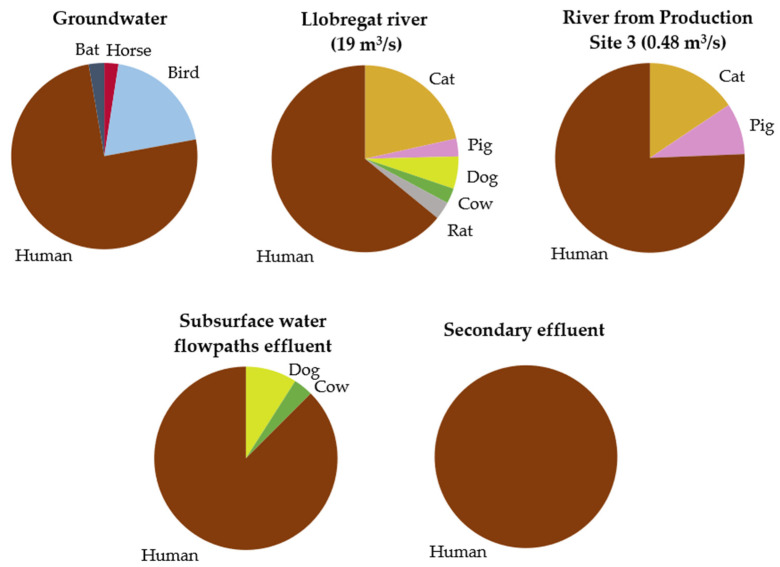
Distribution of vertebrate virus reads among different hosts obtained using target enrichment sequencing from different types of irrigation water sources: groundwater, Llobregat river (flow of 19 m^3^/s), river water from Production Site 3 (flow of 0.48 m^3^/s), subsurface water flow-path effluent and secondary effluent. Further details are provided in Supplementary Materials, Table S2.

All contigs obtained from Genome Detective with nucleotide identity above 70% were further analyzed, resulting in a total of 77,565 viral reads: 31,634 reads for GW1.1, 44 reads for STR1.1, 977 reads for GW2.1, 1609 reads for RIV3.1, 20 reads for LET3.4, 9 reads for PAR3.2, 7446 reads for LLRIV.1, 16,421 reads for SE and 19,405 reads for WFPE. From the negative control included in the analysis, seven reads were assigned to the bacteriophage family *Microviridae*, but no other viral assignments were obtained. Further information about the assignment and distribution of all viral reads obtained in different hosts is detailed in Supplementary Materials, Table S2. Most of the reads belonged to bacteriophages (48.91%), followed by viruses that infect invertebrates (33.95%), plants (8.28%), humans (6.15%), and other vertebrates (2.44%). Using this capture approach, reads assigned to vertebrate viruses were obtained from all irrigation water samples, with the highest diversity found in a Llobregat river water sample (LLRIV.1). The distribution of vertebrate virus reads obtained using TES from different types of irrigation water sources is shown in Figure 2. Of the obtained reads of viruses that infect vertebrates, human viruses were present in greater proportions and accounted for all viruses detected in secondary-treated wastewater. In the Llobregat river, the highest number and diversity of reads were assigned to viruses that infect non-human vertebrates, including feline, canine, porcine, rodent, and cattle viruses belonging to *Astroviridae, Caliciviridae, Parvoviridae*, and *Picornaviridae*. Avian, equine and bat viruses belonging to *Genomoviridae* and *Parvoviridae* were detected in groundwater, whereas river water from Production Site 3 was found to have feline and porcine viral members of *Astroviridae* and *Genomoviridae*. Treated wastewater showed a huge proportion of human virus reads, with a low proportion representing canine and cattle parvoviruses in the effluent of subsurface water flow paths.

In food samples, no reads of viruses that infect humans or other vertebrates were obtained from strawberry and lettuce samples, despite the detection of NoV GII and HAdV by qPCR, respectively, and three reads assigned to a human virus belonging to *Caliciviridae* were obtained from the parsley sample, which showed both HAdV and NoV GII contamination in qPCR assays.

The human pathogenic viruses sequenced using TES are summarized in Table 3. Despite having samples positive for HAdV in the qPCR assay, no assignments belonging to *Adenoviridae* were obtained. Reads assigned to members of *Caliciviridae* were obtained from the Llobregat river and the parsley sample, as mentioned previously. Sequences obtained from the Llobregat river were assigned to NoV GI.1, GII.4, and GII.17, the last of which was also the genotype found in parsley. In this study, all sequences assigned to *Astroviridae* were mainly detected in river water samples. The Llobregat river contained a wide diversity of Human Astroviruses (HAstVs) belonging to different species, including *Mamastrovirus 1* (HAstV-1 and HAstV-4), *Mamastrovirus 6* (AstV-MLB1 and AstV-MLB2), and *Mamastrovirus 8* (HAstV-VA2). HAstV-1 and HAstV-5 were also detected in the river sample from Production Site 3. The presence of HAstV-5 was also detected in subsurface water flow-path effluent. Members of *Picornaviridae* were detected in river water and treated wastewater. Sequences of *Aichivirus A*, typed as Aichi virus 1, were sequenced from river water, whereas *Salivirus A* was found in river water and treated wastewater. Other assignments obtained from irrigation water samples belonged to *Circoviridae,* with sequences detected in groundwater and secondary effluent, and *Parvoviridae,* with sequences detected in groundwater, river water, and subsurface water flow-path effluent.

### 3.3. Diversity of NoV and HPV Sequencing in Irrigation Water and Organic Food Using ADS

NoV sequences obtained using ADS and classified by VP1 analysis are shown in Table 4. Treated wastewater samples (SE and WFPE) presented the highest diversity of NoV GI, whereas the highest diversity of NoV GII was detected in Llobregat river water (LLRIV.1). NoV GI.4 was the most abundant genotype, and it also accounted for the highest number of NoV reads in Llobregat river water. Among NoV GII sequences, NoV GII.4 and GII.13 were the genotypes that appeared in a greater number of samples, and a high number of reads were obtained mainly from Llobregat river water. Other NoV GII genotypes sequenced from the Llobregat river were GII.2, GII.17, and GII.3.

All food samples presented a lower diversity of NoV, but it is remarkable that all NoV genotypes identified were also found in some of the irrigation water sources. NoV GI members found in food samples, GI.4 and GI.1 in strawberry (STR1.1), and GI.4 in parsley (PAR3.2), were also found in river water and treated wastewater. NoV GII genotypes found in parsley samples, GII.13 and GII.2 in PAR3.1 and GII.4 and GII.13 in PAR3.2, were also found in different irrigation water sources.

HPV and other PV sequences obtained using ADS and classified by L1 analysis are shown in Table 5. The sequences obtained were classified into 16 different HPV types, most of which were members of the genus *Betapapillomavirus*. Llobregat river water and treated wastewater showed the highest diversity of HPV. HPV-92, HPV-105, and HPV-122, belonging to the species *Betapapillomavirus 4*, *1*, and *2*, respectively, were sequenced from all samples. Few *Alphapapillomavirus* were identified (HPV-177 and HPV-57), and only one *Gammapapillomavirus* (HPV-4) was found in river and lettuce samples from Production Site 3, in the Llobregat river and in treated wastewater samples (SE and WFPE). Other PVs not classified as HPV were sequenced: *Bos taurus Papillomavirus 7* (BPV-7) from treated wastewater samples and *Rattus norvegicus Papillomavirus 2* (RnPV-2) from a Llobregat river sample (LLRIV.2).

As was the case with NoV, all HPV types detected in food samples were also identified in some of the irrigation waters.

## 4. Discussion

At present, quantitative PCR is the most common method applied to the identification of viral contamination in foods. ISO methods focused on qPCR detection and quantification of NoV and HAV in different food matrices are available and can be applied even when viral food quality is not included in any regulations. Additionally, HAdV has been suggested and proven to be useful as a viral fecal indicator in water matrices, as this human pathogen is widely detected when water is affected by sewage [80,81,82,83] and has been previously detected in conventional and reclaimed irrigation water [29]. In this study, qPCR detected the presence of HAdV in river water and treated wastewater, indicating that irrigation water is a potential source of human fecal contamination and can potentially become a vehicle for viruses that are transmitted via the fecal–oral route through fresh fruits and vegetables. Using the HAdV indicator, fecal contamination was also detected in the parsley, lettuce, and strawberry samples tested in this study. In 2017, Fernández-Cassi et al. also described the presence of this indicator in water samples from the Besòs river and parsley samples irrigated with this river water source [5]. Other studies have reported HAdV in lettuce and irrigation water [84] and in strawberries collected from European food production chains [85].

In addition to HAdV, other pathogenic viruses were detected by qPCR in this study. NoV GI was found in river water and treated wastewater, but it was not detected in food or irrigation water samples from the organic production sites, despite the fact that some were positive for HAdV. Other studies reported NoV GI contamination in strawberries and suspected irrigation water to be the potential source of contamination [86]. On the contrary, NoV GII was widely present in all types of samples. All irrigation water sources and 25% of food samples showed NoV GII contamination in concentrations consistent with those reported in other studies in which polluted water was suspected to be the contamination source of NoV in fresh fruits and vegetables [84,85,87]. Secondary effluent and the effluent of subsurface water flow paths showed similar levels of contamination, indicating that these flow paths were not efficient in eliminating viral contaminants.

HEV was not detected in any of the samples analyzed in this study. Other studies have found HEV to be present in fresh produce: in a pack of frozen raspberries taken from a cold room at point-of-sale [85], in an irrigated, field-grown strawberry sample suspected to be contaminated by irrigation water [86] and in lettuce heads from primary production sites and at point-of-sale [84]. This last study also reported the presence of HEV in irrigation groundwater, which was also described by Rusiñol et al. in an area with intensive pig farming activities [22]. HEV is a zoonotic pathogen that can cause self-limiting or fulminant hepatitis in humans, and it is important to check for its presence since an increasing number of foodborne HEV cases are being reported in Europe, frequently associated with the consumption of pork products [27].

Ultimately, qPCR assays revealed low but frequent contamination associated with human pathogenic viruses in fresh produce and irrigation water, with NoV GII being the principal agent detected. Regarding the NGS data obtained in this study, a targeted assay (TES) was applied by using the VirCapSeqVERT Capture Panel (Roche, Basel, Switzerland) on a selection of samples showing fecal contamination or the presence of specific viral pathogens. This panel employs approximately 2 million biotinylated oligonucleotide probes designed to bind coding sequences of all viral taxa known to infect vertebrates. The TES approach is known to facilitate the detection of vertebrate viruses [68], although some sequences from other viral hosts can be identified [21]. In fact, with the real picture of the virome in mind, vertebrate viruses are commonly found in low proportions with respect to other viruses such as bacteriophages and plant viruses, which have been described as the most abundant in sewage samples [65,88]. TES has been successfully employed in previous studies [21,49,50,67,68] to improve the detection of vertebrate viruses of interest that would otherwise be difficult due to their low concentrations in environmental samples. Recently, the VirCapSeqVERT Capture Panel was applied in a metagenomic study to evaluate NoV genomic diversity in oysters [50], but to date, no studies have applied this capture approach to fresh fruits and vegetables. In this study, TES allowed the acquisition of reads assigned to vertebrate viruses from all irrigation water samples, with the highest diversity observed in the Llobregat river water sample (LLRIV.1). Among the viruses identified in food samples, NoV GII sequences obtained from parsley, which showed a concentration of 7.12 × 10^1^ GC/25 g by NoV GII qPCR, constituted a unique contig that was typed as NoV GII.17. This genotype emerged as a major cause of gastroenteritis outbreaks in China and Japan in the winter of 2014/2015 [89]. Interestingly, NoV GII sequences were not obtained from the strawberry sample STR1.1 using the capture approach despite it showing a high concentration of NoV GII in the specific qPCR assay. This result could be associated with differences in food matrices: detecting viruses in berries is known to be challenging due to the presence of various inhibitory substances and low pH [90]. Bartsch et al. also obtained a low number of NoV reads from the metagenomic analysis of frozen strawberries involved in a large NoV gastroenteritis outbreak [39]. The emergent genotype detected in parsley—NoV GII.17—was also sequenced from the Llobregat river, which also showed other sequences belonging to the *Norwalk virus* typed as NoV GI.1 and NoV GII.4.

ADS assays enabled a better characterization of the NoV diversity present in the studied fresh produce and irrigation water samples. This approach, based on the mass sequencing of traditionally Sanger-sequenced PCR amplicons, facilitates the detailed study of specific families and their diversity within a sample [21] and has previously been applied to environmental and shellfish samples for studying viral groups—such as *Adenoviridae* [21,65,91], *Papillomaviridae* [21,23,92,93], or NoVs [48,52,53]—but none of these studies investigated fresh food samples. Due to differences in the sensitivities of NoV assays and the fact that the concentrations detected were low, not all qPCR-positive samples were positive in RT-PCR amplification for ADS. In particular, STR1.1, PAR3.1, PAR3.2, and RIV3.1, which were qPCR-positive for NoV GII and qPCR-negative for NoV GI, produced an RT-PCR amplicon of the expected size for NoV GI but not for NoV GII. Additionally, groundwater and treated wastewater (WPFE) samples were positive for NoV GII in the qPCR assay, but no amplicons of the expected size were produced by conventional RT-PCR. This result may be explained by the lower sensitivity of this technique as compared to the qPCR assay [87,94]. In contrast, LET3.4 showed an amplicon of the expected size for NoV GI, although it did not show (or at least not above the limit of detection) NoV contamination by qPCR, which could be due to sensitivity differences between assays when concentrations are around this limit.

NoV GII.4 has been reported to be the most predominant genotype worldwide [95,96]. This is consistent with our findings, in which the presence of the GII.4 genotype was widely observed in different samples using ADS, including river water and treated wastewater samples, and also in a parsley sample. Llobregat river water was the sample with the highest number of sequences obtained from this specific genotype using ADS, and it was also the only sample in which this genotype was possible to sequence using TES. In fact, the three genotypes identified from the Llobregat river using TES, GI.1, GII.4, and GII.17, were also identified by ADS. It is important to highlight that all NoV genotypes found in food samples, GI.4 and GI.1 in strawberry and GI.4, GII.4, GII.13, and GII.2 in parsley, were also found in irrigation water samples. Maunula et al. also detected NoV GI.4 in frozen raspberries from one particular batch that was confirmed to be the source of a described cluster of NoV outbreaks affecting about 200 people in Southern Finland in 2009 [97].

In addition to viruses belonging to *Caliciviridae,* the TES results also showed other pathogenic or potentially pathogenic viruses present in river water, specifically viruses belonging to *Astroviridae*, *Picornaviridae*, and *Parvoviridae* families, which is consistent with the virome previously described in a river in the same geographical area that is also used for irrigation [5]. The Llobregat river contained a wide diversity of HAstVs, including the species *Mamastrovirus 1*, *6*, and *8.* HAstVs are important agents causing acute gastroenteritis in children and have been involved in outbreaks affecting adults [95,98]. Although HAstV prevalence seems to be of lower importance compared to the number of outbreaks caused by NoV, its importance might be underestimated [5]. Recently, HAstVs have also been associated with other pathologies, such as meningitis and acute flaccid paralysis [95,99,100,101]. HAstV-1, which was found in river water samples, is the most common type associated with infantile gastroenteritis [95,102]. *Picornaviridae* sequences found in river water samples belonged to the genus *Kobuvirus,* specifically to *Aichivirus A,* a viral species that includes members recognized as human pathogens that cause gastroenteritis outbreaks [103,104,105]. Other *Picornaviridae* sequences were assigned to *Salivirus A,* belonging to the genus *Salivirus.* The highest proportion of the vertebrate virus sequences obtained were human viruses, and among all types of irrigation water, the Llobregat river showed the highest number of reads and diversity of viruses that infect non-human vertebrates, including feline, canine, porcine, rodent, and cattle viruses belonging to *Astroviridae, Caliciviridae, Parvoviridae*, and *Picornaviridae* families. River water from Production Site 3 presented feline and porcine viruses designated as *Astroviridae* members. Among all vertebrate virus sequences obtained using TES, the greatest proportion of viruses that infect humans was observed, as expected, in treated wastewater, with human viruses accounting for all viruses detected in secondary-treated wastewater, and were identified as members belonging to *Astroviridae*, *Circoviridae*, *Parvoviridae*, and *Picornaviridae*, which is consistent with other metagenomic studies [22,106]. The non-human vertebrate viruses sequenced in treated wastewater were canine and cattle parvoviruses. Human viral sequences belonging to *Circoviridae* and *Parvoviridae* and avian, equine and bat viruses belonging to *Genomoviridae* and *Parvoviridae* were also detected in groundwater samples. Viruses are commonly detected in groundwater used for irrigation, even though it is often considered a microbially safe source [7,107]. These viruses may originate from the leakage of sewage water or diffuse contamination from livestock production zones close to produce fields [108]. In this study, the sequences obtained using TES assigned to viruses that infect non-human vertebrates of interest could be related to intensive farming activities in the surrounding areas, which have the potential to contaminate irrigation water through leakage. Despite lower yields of production in organic farming compared with intensive agriculture, organic farming systems represent an attractive environmentally friendly alternative, delivering equally or more nutritious foods that contain less, or no, pesticide residues [109]. This system relies on the incorporation of organic material into the soil using animal manure as fertilizer [110,111,112]. Although animal manure is considered a beneficial organic fertilizer and a good source of nutrients, it is also a well-known source of foodborne pathogenic bacteria, parasites, and viruses if it is not adequately aged or treated before application [13,113,114] and, therefore, is a potential consumption risk.

Despite having samples positive for HAdV in the qPCR assay, no assignments belonging to *Adenoviridae* were obtained using TES. It has been previously reported that the SISPA protocol, which is performed prior to library preparation in order to overcome the limitation of low quantities of viral genomes, might introduce bias by amplifying the most abundant genomes and, therefore, underrepresenting others [115,116]. Fernández-Cassi et al. already noted these specific difficulties when detecting low numbers of dsDNA viruses such as HAdV in river water or when unable to detect HAdV in parsley samples that were positive for the virus in the qPCR assay [5]. Considering that TES did not allow the acquisition of HAdV sequences, a more suitable approach, such as ADS, should be applied to improve the sensitivity of viral metagenomics. Similarly, no sequences of *Papillomaviridae* were obtained from the TES approach. The detection of HPVs could also be affected by SISPA bias due to their dsDNA genome. However, ADS was demonstrated to be a suitable HPV detection and diversity exploration tool that overcomes this specific TES limitation since a wide variety of HPV was observed using this approach. Two different primer sets were used: GP5+/GP6+ were designed for detecting mucosal HPV types [79], whereas FAP6085/6319 were designed for detecting cutaneous HPV types [78]. The role of cutaneous HPV types in pathogenesis remains unclear: some are regarded as potential high-risk types because they are found in squamous cell carcinoma (SCC), such as HPV-5 or HPV-8, while most other cutaneous HPVs are only associated with benign lesions such as epidermodysplasia verruciformis [117,118]. Most of the HPV types sequenced in this study were classified as *Betapapillomavirus.* River and treated wastewater samples contained the highest diversity of *Betapapillomavirus:* HPV-12, HPV-17, HPV-37, HPV-38, HPV-76, HPV-92, HPV-96, HPV-105, HPV-122, HPV-145, and HPV-182. Treated wastewater also contained HPV-196 and HPV-8 sequences, the last of which is the HPV mostly found in SCC of the skin [117], and it has been described as an abundant HPV type in urban sewage [21]. HPV-12, HPV-37, HPV-38, HPV-76, HPV-92, HPV-105, HPV-122, and HPV-145 types were also identified in lettuce and parsley samples, and HPV-96 was only detected in lettuce. HPVs belonging to *Alphapapillomavirus* were also sequenced. HPV-177 was identified in river water and treated wastewater, as well as in lettuce and parsley samples. HPV-57 is associated with common warts typically occurring in the anogenital region [119] and was identified in river water, treated wastewater, and lettuce. Members belonging to the genus *Alphapapillomavirus* have been described previously in raw sewage [21,23]. HPV was frequently detected in the food and irrigation water samples analyzed in this study and should therefore be considered a potential emergent pathogen, and the role of irrigation water and fresh produce in the transmission of HPV should be further investigated. To our knowledge, this study is the first to provide data on the diversity of HPVs and NoVs present in fresh fruit and vegetable samples. Manipulation during the production process should not be ruled out as a route through which viral contamination is introduced to the food analyzed in this study. It is possible that some of the HPVs detected in food samples were skin contaminants, since most of the reads were classified as members belonging to the genus *Betapapillomavirus*, which includes types commonly isolated from skin. From sample collection to analysis, samples were handled carefully using gloves during all processes in order to avoid any kind of viral contamination. It is important to remark that all viruses detected in food were also detected in water samples, supporting the possibility that irrigation water is a source of contaminant HPVs.

The results of this study indicate that irrigation water and fresh fruits and vegetables present a wide variety of viral pathogens that may pose a risk to humans. Natural water treatments or disinfection procedures are often necessary to obtain high quality irrigation water. The implementation of quality monitoring programs integrated with Quantitative Microbial Risk Assessment (QMRA) investigations will provide an estimate of the level of risk and the treatments needed to produce high quality water. The NGS techniques described in this study would be useful for the identification of significant pathogens present in water and food for risk assessment studies, the selection of water treatments and the development of optimized site-specific safety plans. Because wastewater is usually of poor chemical and microbiological quality, extensive treatments are required before it can be safely used for irrigation [113]. However, drought conditions are linked to the utilization of untreated wastewater or contaminated groundwater for irrigation due to the lack of clean water, increasing the chances of microbial contamination of food products and soil [120]. In addition, due to climate change, unpredictable meteorological phenomena are expected to become more frequent and likely to drive an increase in the incidence of water scarcity with a probability of drought [1], potentially leading to the utilization of reclaimed water as a common source for irrigation in many locations. The association between pathogen diversity in this type of irrigation water and foodborne disease would imply that efficient treatments must be implemented to promote its use as an additional and safe water source. This study only suggests a possible link between irrigation water and the contamination origin of fresh produce, but other contamination factors involved in agricultural practices could certainly have an impact and should be considered, such as organic fertilizer or run-off from nearby animal pastures, which could also be vehicles for viral contamination [85,121]. Furthermore, detection of viral genomic sequences does not necessarily imply a consumption risk since infectivity potential was not verified in this study. In sum, irrigation water and animal manure are considered the two most important pathways of pathogen transmission from human or animal hosts to fresh produce at the preharvest level [10].

Checking for the presence of viruses as a part of controlling the quality of irrigation water is a key intervention step to reduce the risk of transferring contamination to fresh produce [108,122]. Further studies are needed to confirm and elucidate the significance of the information provided in terms of risk for consumption. From the data obtained in this study, we conclude that TES is a useful tool to obtain a broad picture of vertebrate viruses that integrate into the virome of irrigation water samples, whereas ADS allows the in-depth characterization of the diversity of a specific viral pathogen contaminating irrigation water and fresh produce. More relevant is that, by applying these methodologies, human pathogenic viruses were detected in samples in which qPCR showed low levels of contamination, suggesting that NGS approaches could be a suitable tool to identify and characterize viral pathogens and improve food monitoring and foodborne disease outbreak control.

## 5. Conclusions

- The fresh fruits and vegetables cultured in the analyzed organic farms show a high frequency of viral contamination, and the contaminating viruses are also detected in river water and tertiary effluents from subsurface water flow paths used in irrigation, indicating that they are common circulating pathogens.
- In this study, irrigation water is the most probable source of the viral pathogens, primarily NoV and HPV, detected in food. All pathogens were detected in some types of irrigation water, such as river water or treated wastewater, indicating that are potential sources of contamination. However, virus infectivity potential was not analyzed in this study.
- A wide diversity of cutaneous HPV was detected in fresh produce and a wider diversity of HPV was identified in more polluted water samples, such as river and treated wastewater samples. HPV-8, a high-risk type associated with SCC, was found in treated wastewater.
- The three methodologies used for the analysis of viral contamination of irrigation water and organic food are useful and produce different types of information: (a) qPCR is a highly sensitive quantitative and specific technique, (b) TES shows the presence of relatively abundant viral pathogens present in irrigation water, including unexpected viral pathogens and potentially zoonotic strains, and (c) ADS provides higher sensitivity for the identification of viral types or variants in viral groups that contaminate food and water.

## Figures and Tables

**Table 1:** Irrigation water (GW: groundwater; RIV: river), lettuce (LET), strawberry (STR), and parsley (PAR) samples collected from Production Sites 1, 2, and 3 and also alternative irrigation waters, i.e., Llobregat river water (LLRIV) and treated wastewater (SE: secondary effluent; WFPE: subsurface water flow-path effluent), included in this study.

	Irrigation Water	Lettuce	Strawberry	Parsley
**Production Site 1**	GW1.1 to GW1.4	LET1.1 to LET1.4	STR1.1 to STR1.8	PAR1.1 to PAR1.4
**Production Site 2**	GW2.1 to GW2.4	LET2.1 to LET2.4		
**Production Site 3**	RIV3.1 to RIV3.4	LET3.1 to LET3.4	STR3.1 to STR3.4	PAR3.1 to PAR3.4
**Llobregat river**	LLRIV.1 and LLRIV.2			
**Treated wastewater**	SE and WFPE			

**Table 2:** Viral concentrations obtained from alternative irrigation water sources. Values are expressed in GC/L. ND: not detected. SE: secondary effluent, WFPE: subsurface water flow-path effluent.

		HAdV	NoV GI	NoV GII	HEV
**Llobregat river**	LLRIV.1	3.47 × 10^4^	6.36 × 10^3^	1.33 × 10^4^	ND
	LLRIV.2	5.99 × 10^3^	1.01 × 10^4^	4.75 × 10^4^	ND
**Treated wastewater**	SE	8.99 × 10^4^	3.77 × 10^3^	ND	ND
	WFPE	1.88 × 10^5^	9.49 × 10^3^	6.64 × 10^2^	ND

**Table 3:** Human viruses sequenced in irrigation water from Production Site 1, Production Site 2, and Production Site 3, alternative irrigation water sources and parsley from Production Site 3 analyzed using target enrichment sequencing.

Family	Genus	Species	Genogroup/ Genotype/ Serotype	Samples					
				Contigs	Length (bp)	Nucleotide Identity (%)	Genome Coverage (%)	Sample Name	Site
*Astroviridae*	*Mamastrovirus*	*Mamastrovirus 1*	HAstV-1	2	429–435	98.60–99.07	6.3–6.4	RIV3.1	Production Site 3
				1	551	98.19	8.1	LLRIV.1	Llobregat river
			HAstV-4	2	800–3071	92.10–93.62	11.9–45.7	LLRIV.1	Llobregat river
			HAstV-5	1	2869	92.36	42.2	RIV3.1	Production Site 3
				1	808	97.65	12.1	WFPE	Treated wastewater
		*Mamastrovirus 6*	AstV-MLB1	1	420	95.00	6.8	LLRIV.1	Llobregat river
			AstV-MLB2	1	364	98.35	5.9	LLRIV.1	Llobregat river
		*Mamastrovirus 8*	HAstV-VA2	1	585	99.49	9.0	LLRIV.1	Llobregat river
*Caliciviridae*	*Norovirus*	*Norwalk virus*	NoV GI.1	1	524	90.15	6.8	LLRIV.1	Llobregat river
			NoV GII.4	2	689–845	92.79–97.98	9.1–11.2	LLRIV.1	Llobregat river
			NoV GII.17	1	282	97.28	3.8	PAR3.2	Production Site 3
				2	632–717	95.66–98.58	8.4–9.5	LLRIV.1	Llobregat river
*Circoviridae*	*Cyclovirus*	*Human associated cyclovirus 6*	NG12	1	1541	81.00	85.9	GW1.1	Production Site 1
				1	433	88.29	24.10	SE	Treated wastewater
*Parvoviridae*	*Dependoparvovirus*	*Adeno-associated dependoparvovirus A*	AAV2	1	124	95.97	2.7	GW2.1	Production Site 2
				1	3040	97.27	64.9	RIV3.1	Production Site 3
				3	932–1216	96.46–98.39	19.9–26.0	LLRIV.1	Llobregat river
				1	1438	96.45	30.7	WFPE	Treated wastewater
			AAV6	1	682	79.71	14.6	LLRIV.1	Llobregat river
	*Bocaparvovirus*	*Human bocavirus 3*	Undetermined	3	342–566	95.61–98.25	6.6–10.8	LLRIV.1	Llobregat river
*Picornaviridae*	*Kobuvirus*	*Aichivirus A*	Aichi virus 1	4	348–724	94.31–99.42	4.2–8.8	RIV3.1	Production Site 3
				5	404–952	95.79–97.56	4.9–11.5	LLRIV.1	Llobregat river
	*Salivirus*	*Salivirus A*	Undetermined	3	683–1197	97.21–98.41	8.7–15.3	RIV3.1	Production Site 3
				2	459–552	97.67–98.04	5.9–7.0	LLRIV.1	Llobregat river
				2	395–907	96.73–97.97	5–11.6	SE	Treated wastewater
				3	516–623	97.16–97.87	6.6–7.9	WFPE	Treated wastewater

**Table 4:** Number of reads of each NoV genotype obtained from a strawberry sample from Production Site 1, irrigation water and parsley samples from Production Site 3 and alternative irrigation water sources using amplicon deep sequencing.

		Production Site 1	Production Site 3			Llobregat River		Treated Wastewater	
		STR1.1	RIV3.1	PAR3.1	PAR3.2	LLRIV.1	LLRIV.2	SE	WFPE
NoV GI	GI.4	17	1		10	98,699	3	15	59
	GI.1	4	2			15		23,411	35,901
	GI.2					8	28	2	2
	GI.3					4		9	9500
	GI.5		2					12,021	475
NoV GII	GII.4		1		3	9331		3	6
	GII.13		2	3	11	49,651			16
	GII.2			2		16,933			4
	GII.17					52	1203		
	GII.3					68			
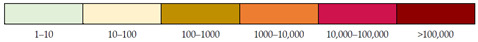									

**Table 5:** Number of reads of each type of human and other Papillomavirus obtained from irrigation water, lettuce, and parsley samples from Production Site 3 and alternative irrigation water sources using amplicon deep sequencing.

		Production Site 3				Llobregat River		Treated Wastewater	
		RIV3.1	LET3.4	PAR3.1	PAR3.2	LLRIV.1	LLRIV.2	SE	WFPE
HPV	HPV-92	1	2	14	6	39,158	15	3	16
	HPV-105	3	45	381,617	16	108	197	13	337,323
	HPV-122	2	19	90	50	271,741	38	11	114
	HPV-38		152,893	14	19	23	1161	8	7
	HPV-145		102,028	17	8	8	150	5	6
	HPV-177	16	10	3		10	20	8	28
	HPV-182	1	9	580		1	1	3	2
	HPV-37		101		45	8	9	90	19
	HPV-76		84	6		8	87,512	4	10
	HPV-96		2			1	145	4	1171
	HPV-4	7	1				2	169,019	3
	HPV-57	6	56					76,118	1
	HPV-12		20	16		79	152,261	17	35
	HPV-8							321	1
	HPV-17						2		3
	HPV-196						49	1	
Other PV	BPV-7							1	1
	RnPV-2						17		
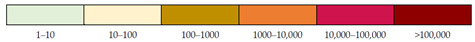									

## Data Availability

The datasets generated during the current study are available in Zenodo under the DOI number https://doi.org/10.5281/zenodo.4722797.

## Supplementary Materials

The following are available online at https://www.mdpi.com/article/10.3390/foods10081820/s1, Table S1: Quantification of viral pathogens in irrigation water and fresh produce samples. Values are expressed in GC/L for irrigation water samples and in GC/25 g for food samples. ND: Not Detected; Table S2: Reads obtained from each viral assignment in irrigation water, strawberry, lettuce, and parsley samples using target enrichment sequencing. Colors represent the specific host of vertebrate viruses, legend can be found under the table.
