# Supplementary material for: NGS Techniques Reveal a High Diversity of RNA Viral Pathogens and Papillomaviruses in Fresh Produce and Irrigation Water
Source: Foods. 2021 Aug 6;10(8):1820. doi: 10.3390/foods10081820 (PMC8394881; doi:10.3390/foods10081820)
Supplement: Supplementary file 1 [file foods-10-01820-s001.zip › foods-1229021-Supplementary.pdf]

foods

Supplementary Files

Table S1. Quantification of viral pathogens in irrigation water and fresh produce samples. Values are expressed in GC/L for irrigation water samples and in GC/25 g for food samples. ND: Not Detected.

		HAdV	NoV GI	NoV GII	HEV
Production Site 1	GW1.1	ND	ND	2.37×10^2	ND
	GW1.2	ND	ND	2.51×10^2	ND
	GW1.3	ND	ND	ND	ND
	GW1.4	ND	ND	ND	ND
	LET1.1	ND	ND	ND	ND
	LET1.2	ND	ND	ND	ND
	LET1.3	ND	ND	ND	ND
	LET1.4	ND	ND	4.35×10^2	ND
	STR1.1	ND	ND	3.26×10^2	ND
	STR1.2	ND	ND	2.55×10^2	ND
	STR1.3	ND	ND	ND	ND
	STR1.4	ND	ND	ND	ND
	STR1.5	ND	ND	ND	ND
	STR1.6	ND	ND	ND	ND
	STR1.7	ND	ND	ND	ND
	STR1.8	ND	ND	ND	ND
	PAR1.1	6.05×10^2	ND	ND	ND
	PAR1.2	ND	ND	ND	ND
	PAR1.3	ND	ND	ND	ND
	PAR1.4	ND	ND	ND	ND
Production Site 2	GW2.1	ND	ND	9.02×10^0	ND
	GW2.2	ND	ND	1.96×10^2	ND
	GW2.3	ND	ND	ND	ND
	GW2.4	ND	ND	ND	ND
	LET2.1	1.40×10^1	ND	ND	ND
	LET2.2	ND	ND	ND	ND
	LET2.3	3.02×10^2	ND	ND	ND
	LET2.4	ND	ND	3.35×10^2	ND
Production Site 3	RIV3.1	ND	ND	1.34×10^1	ND
	RIV3.2	ND	ND	ND	ND
	RIV3.3	ND	ND	ND	ND
	RIV3.4	ND	ND	ND	ND
	LET3.1	1.03×10^2	ND	ND	ND
	LET3.2	ND	ND	ND	ND
	LET3.3	ND	ND	ND	ND
	LET3.4	1.10×10^2	ND	ND	ND
	STR3.1	ND	ND	ND	ND
	STR3.2	4.16×10^1	ND	5.60×10^2	ND
	STR3.3	5.27×10^1	ND	ND	ND
	STR3.4	ND	ND	ND	ND
	PAR3.1	ND	ND	2.44×10^1	ND
	PAR3.2	8.83×10^2	ND	7.12×10^1	ND
	PAR3.3	8.76×10^2	ND	9.68×10^0	ND
	PAR3.4	ND	ND	9.64×10^0	ND
Llobregat river	LLRIV.1	3.47×10^4	6.36×10^3	1.33×10^4	ND
	LLRIV.2	5.99E+03	1.01×10^4	4.75×10^4	ND
Treated wastewater	SE	8.99×10^4	3.77×10^3	ND	ND
	WFPE	1.88×10^5	9.49×10^3	6.64×10^2	ND

foods

MDPI

Table S2. Reads obtained from each viral assignment in irrigation water, strawberry, lettuce and parsley samples using target enrichment sequencing. Colors represent the specific host of vertebrate viruses, legend can be found under the table.

			Production Site 1		Production Site 2	Production Site 3			Llobregat river	Treated wastewater	
**Host**	**Family**	**Assignment**	**GW1**	**STR1.1**	**GW2**	**RIV3**	**LET3.4**	**PAR3.2**	**LLRIV.1**	**SE**	**WFPE**
Bacteria	*Inoviridae*	Uncultured phage WW-nAnB			12				49		
	Leviviridae	Acinetobacter phage AP205				3					
		Escherichia virus MS2			90	62		4	6		18,808
	Microviridae	Bdellovibrio virus MH2K							10		
		Escherichia virus phiX174					4	2	4		
		Gokushovirinae Bog1183_53							2		
		Gokushovirinae GAIR4	18,239	18	38				53		22
		Gokushovirinae GNX3R			7						
	Myoviridae	Salmonella phage RE-2010		4							
		Salmonella virus Fels2		16							
	Podoviridae	Uncultured crAssphage									2
	Siphoviridae	Salicola phage CGphi29				14					
		Salmonella virus SP31			61						
		Serratia phage Eta			385						
		Streptococcus virus Sfi19							20		
Human	*Astroviridae*	Astrovirus MLB1							4		
		Astrovirus MLB2							6		
		HMO Astrovirus A							18		
		Mamastrovirus 1				255			752		24
	Caliciviridae	Norovirus GI							20		
		Norovirus GII						3	115		
	Circoviridae	Human associated cyclovirus 6	2,026							18	

	Parvoviridae	Adeno-associated virus - 2			3	187			573		89
		Human bocavirus 3							62		
	Picornaviridae	Aichivirus A				73			155		
		Salivirus A				159			40	101	90
Invertebrates	*Dicistroviridae*	Aphid lethal paralysis virus	2,013	2	8	135				3,798	
		Big Sioux River virus	18								
		Kashmir bee virus	437		10						
		Rhopalosiphum padi virus		4						11,283	
	Genomoviridae	Tick associated genomovirus 3	8,254								
	Iflaviridae	Sacbrood virus	24			14					
		Varroa destructor virus 1	28								
	Iridoviridae	Invertebrate iridescent virus 31				4					
	Parvoviridae	Anopheles gambiae densovirus							6		
		Dipteran ambidensovirus 1							28		
		Junonia coenia densovirus				4					
	Unclassified	Giant house spider associated circular virus 2			190						
		Odonata-associated circular virus-18									18
	Unclassified *Picornavirales*	Biomphalaria virus 2							2		
	Unclassified *Riboviria*	Beihai permutotetra-like virus 2	2								
		Changjiang picorna-like virus 14				10					
		Hubei picorna-like virus 15				8				28	
		Hubei picorna-like virus 51				8					
Other Vertebrates	*Astroviridae*	Feline astrovirus 2				139			586		
		Porcine astrovirus 4				54					
	Caliciviridae	Sapovirus GIII							16		
	Genomoviridae	Equine associated gemycircularvirus 1	66								
		Gerygone associated gemycircularvirus 1			4						
		Gerygone associated gemycircularvirus 2	503								
		Pteropus associated gemycircularvirus 2			73						
		Sewage derived gemykibivirus 1				24					

	Parvoviridae	Carnivore bocaparvovirus 2							152		21
		Parus major densovirus	24								
		Porcine bocavirus 3							12		
		Porcine bocavirus 4-1							50		
		Porcine parvovirus							6		
		Primate bocaparvovirus 2							65		8
		Rodent protoparvovirus 1							76		
		Ungulate bocaparvovirus 4							7		
	Picornaviridae	Norway rat hunnivirus							8		
Plant	*Alphaflexiviridae*	Pepino mosaic virus							10		
	Rhabdoviridae	Lettuce big-vein associated varicosavirus					14				
	Tombusviridae	Cucumber necrosis virus							12		
		Moroccan pepper virus			6						
		Tobacco necrosis virus D				4					
		Tomato bushy stunt virus			4						
	Unclassified Riboviria	Tobacco virtovirus 1				11					
	Virgaviridae	Bell pepper mottle virus							4		
		Cucumber green mottle mosaic virus				153			27	14	13
		Pepper mild mottle virus				138			1,404	172	44
		Tobacco mosaic virus							4		
		Tomato mosaic virus				52	2		3,082	984	266
Unknown	*Genomoviridae*	Sewage derived gemycircularvirus 1			80						
		Sewage derived gemykibivirus 2			6						
	Unclassified	Sewage-associated circular DNA virus-20								23	
		Sewage-associated circular DNA virus-27				98					

Human Cat Pig Horse Bird Bat Dog Cow Rat
